# Syndemics and clinical impact of HIV and mental health conditions among people living with HIV: a systematic review and meta-analysis

**DOI:** 10.3389/fpubh.2026.1778334

**Published:** 2026-04-10

**Authors:** Atitegeb Abera Kidie, David Serunjogi, Solomon Moges Demeke, Birtukan Gizachew Ayal, Innocent Mwombeki, Bathsheba Mahenge, Ritah Namugumya, Tiffany E. Gooden, Francis Xavier Kasujja, Mkhoi L. Mkhoi

**Affiliations:** 1 Department of Public Health, College of Health Sciences, Woldia University, Woldia, Ethiopia; 2 MRC/UVRI and LSHTM Uganda Research Unit, Entebbe, Uganda; 3 Department of Psychiatry, College of Health Science, Woldia University, Woldia, Ethiopia; 4 Research, Training and Consultancy Unit, Mirembe National Mental Health Hospital, Dodoma, Tanzania; 5 Department of Psychiatry and Mental Health, University of Dodoma, Dodoma, Tanzania; 6 Makerere University-John Hopkins University (MUJHU) Research Collaboration, Kampala, Uganda; 7 Department of Applied Health Sciences, University of Birmingham, Birmingham, United Kingdom; 8 Global Health and Migration Unit, Department of Women's and Children's Health, International Maternal and Child Health, Uppsala University, Uppsala, Sweden; 9 Department of Microbiology and Parasitology, University of Dodoma, Dodoma, Tanzania

**Keywords:** HIV, mental health, psychosocial factors, syndemic theory, synergising factors

## Abstract

**Introduction:**

People living with HIV (PLWH) may be exposed to harmful political, social, economic and environmental factors that exacerbate their risk of mental health conditions. Such factors can interact synergistically to worsen HIV and mental health-related outcomes, creating a syndemic. This study aims to review existing literature on mental health-related syndemics and their impact on HIV and mental health outcomes.

**Methods:**

CINAHL, Embase, MEDLINE, PsycInfo, Scopus and ProQuest were searched. We included observational studies that investigated a potential mental health-related syndemic and/or reported the impact of a syndemic on HIV outcomes (antiretroviral therapy [ART] adherence or viral suppression), or mental health outcomes (mental health-related quality of life, depression, anxiety, schizophrenia, bipolar disorder, post-traumatic stress disorder or psychological distress) among PLWH. Screening, data extraction and quality assessment were conducted by two independent reviewers. The Newcastle-Ottawa Scale (NOS) was used to assess the quality and risk of bias. The impact of syndemic count on ART adherence and viral suppression was pooled using random effects using STATA and the remaining findings were synthesised narratively. PRISMA guidelines were followed.

**Results:**

32 studies were included with sample sizes ranging from 51 participants to 14,261. Six studies reported on mental health-related syndemics among PLWH, four of which found depression or distress to be the most influential syndemic factor. Mental health conditions within syndemics often cluster and are significantly associated with socioeconomic factors such as food insecurity, stigma and violence. Fifteen of 16 studies found a significant association between adherence and the number of mental health-related syndemics. Pooled odds ratio of seven studies showed a significant reduction in adherence (*OR* = 0.73; 95% *CI* = 0.55 – 0.96); heterogeneity was high (*I*^2^ = 98.58%). Eleven of 13 studies found a significant association between the number of mental health-related syndemics and being virally suppressed. Four studies resulted in a significant pooled odds ratio for having detectable viral load (*OR* = 1.26; 95% *CI* = 1.10 - 1.44); heterogeneity was moderate (*I*^2^ = 52.38%).

**Conclusion:**

Despite wide variation in how syndemics were defined and measured across studies, our findings suggest that mental health conditions, particularly depression, strongly influence synergising syndemics among PLWH, and mental health-related syndemics negatively impact ART adherence and viral load. These findings underscore the need for syndemic-informed holistic care models to address the intersecting burden of mental health conditions and psychosocial factors among PLWH.

## Introduction

There are around 40 million people living with HIV (PLWH) worldwide (1). Compared to people without HIV, PLWH have an increased risk for developing non-communicable diseases, including mental health conditions of depression, anxiety, schizophrenia, bipolar disorder, and post-traumatic stress disorder (PTSD) (2, 3). This heightened vulnerability for developing mental health conditions is in part due to the complex biological reaction caused by the virus and some antiretroviral therapy (ART) (4–6). However, poor mental health among PLWH is also due to the association between HIV and unique psychosocial factors that are increased among PLWH, including stigma, violence, political, legal and social discrimination, precarious employment, and extreme poverty (4). Such factors may create a syndemic, which can be defined as a set of intertwined and mutually enhancing problems that interact and undermine one's wellbeing and increase the risk of negative health outcomes (7).

Syndemic theory was first described and defined following the recognition that HIV, substance use and violence commonly co-occur and were strongly influenced by harmful political, economic and social factors (7, 8). Identifying the syndemics related to HIV and mental health conditions may enable a deeper understanding of the context that perpetuates ill health and poor health outcomes among vulnerable and disadvantaged PLWH. As a response, we can target the wider psychosocial issues that influence and exacerbate HIV and mental health outcomes. For HIV, important outcomes include adherence to ART and viral suppression, both of which are imperative for PLWH to live long, healthy lives and reduce transmission. PLWH with poor mental health are less likely to adhere to ART and achieve viral suppression (9). Thus, understanding when and how HIV and mental health interact will enable the development of targeted interventions to improve wellbeing and physical health.

Existing reviews that focus on HIV-mental health syndemics were not systematically conducted (10–12). Additionally, they either focus on specific populations of PLWH, such as mothers or caregivers, are restricted to a single database search, have limited publication dates, or are not detailed enough in their research questions or findings. To understand the wider evidence base of mental health syndemics among PLWH, this systematic review aims to identify mental health-related syndemics among PLWH, and understand their impact on HIV and mental health outcomes. By synthesising the existing evidence on mental health-related synergies among PLWH, this review aims to enable and encourage more research on how to address the root causes of poor mental health and poor HIV outcomes and further investigate and understand existing syndemics in places where evidence is lacking.

## Materials and methods

This study adheres to the Preferred Reporting Items for Systematic Reviews and Meta-Analyses (PRISMA) guidelines (13) and was registered in the International Prospective Registration Database for Systematic Reviews (PROSPERO) on 18^th^ June 2025, with registration number CRD420251062411. This review aimed to answer the following three research questions:

1. What mental health-related syndemics exist among PLWH and in what context?
2. What is the impact of mental health-related syndemics on ART adherence and viral load?
3. What is the impact of syndemics among PLWH on mental health-related quality of life, depression, anxiety, PTSD, psychological distress, schizophrenia and bipolar disorder?

### Eligibility criteria

All research questions applied the same eligibility criteria to the population: studies that included PLWH only were eligible. For the first research question, studies were eligible if they investigated any potential syndemic specifically using syndemic theory where depression, anxiety, schizophrenia, bipolar disorder, PTSD or psychological distress was included as part of the potential syndemic. Although psychological distress is not technically a mental health condition, it can often be a precursor to depression and anxiety, making it an important element of mental health to target for prevention (14, 15). Also to be eligible for question 1, statistical outputs had to be in line with syndemic theory (multiplicative or additive interaction terms, within a regression, with a post-calculation of the relative excess risk due to the interaction, synergy index or attributable proportion) (16) or confirm a clustering effect of the syndemic (latent class analysis, cluster analysis, network analysis, latent profile analysis, factor analysis, or other person- or variable-centred methods).

For research question 2, studies were included if they (a) specifically used syndemic theory, (b) included depression, anxiety, schizophrenia, bipolar disorder, PTSD or psychological distress within the syndemic and (c) investigated the impact of a syndemic count or syndemic cluster/group on ART adherence or viral suppression. For research question 3, studies were included if they (a) specifically used syndemic theory and (b) investigated the impact of a syndemic count or syndemic cluster/group on depression, anxiety, schizophrenia, bipolar disorder, PTSD, psychological distress or mental health quality of life.

Only cross-sectional, case-control and cohort studies were included. Mixed-methods studies were included only if the quantitative component fit all other eligibility criteria; the qualitative component were not used. Dissertations and PhD theses were eligible for inclusion. Reviews, trials, qualitative studies, abstracts, case series and case reports were excluded from this review.

### Search strategy

We used the PECOS framework to inform our search strategy. The search of six databases was conducted between 11th and 13th June 2025, without applying date or language restrictions. This included the Cumulative Index to Nursing and Allied Health Literature (CINAHL), Embase via Ovid, MEDLINE, PsycInfo, Scopus, and ProQuest Dissertations & Theses Global. A combination of keywords and database-specific subject heading terms was used. The full search strategy and resulting hits from each database are included in our Supplementary material.

Following the formal search described above, we also conducted an informal search by assessing the bibliography of any related review (found within the formal search) for any studies that may have been missed in the formal search.

### Selection process

All search results were imported into Covidence with duplicates removed automatically before screening. Two reviewers (FXK and MLM) independently first screened all titles and abstracts followed by an assessment of any relevant full-text articles against eligibility criteria. Any disagreements were resolved through discussion or consultation with a third reviewer (TEG) where necessary. The selection process was managed within Covidence.

### Data extraction

Data were extracted independently by two reviewers (SMD and DS) using a piloted data extraction tool in Excel created by the research team. The tool included the following elements: study characteristics (title, authors, publication year, conflicts of interests), methods (study design, setting, study period, inclusion and exclusion criteria, aim of the study and follow up time if appropriate), participants (number of included participants, age, sex, ethnicity or similar and other characteristics reported), syndemic factors (definition and measurement of each), outcome measurements (outcome definition and measurement, statistical approach) and results (all relevant statistical outputs and author interpretation). Any disagreements between the two reviewers were resolved through discussion or by a third reviewer (TEG).

### Risk of bias assessment

The risk of bias was assessed using the Newcastle-Ottawa Scale (NOS) (17). Although the NOS is designed for evaluating cohort and case-control studies, the tool is comprehensive and widely supported for use of evaluating cross-sectional studies as well, offering structured assessment across key domains including confounding, selection of participants, classification of exposures, and measurement of outcomes. We used a recently published adaptation of the NOS for cross-sectional studies (18).

Each included study was independently assessed by at least two reviewers (AAK, IM, and BA), with discrepancies resolved through discussion or a third reviewer (TEG). Risk of bias judgments were made per study, and each were given up to 9 stars, where a higher number of stars was indicative of a lower risk of bias and a lower number of stars was indicative of a higher risk of bias.

Certainty of findings was not assessed since the review did not assess the effectiveness of an intervention. Similarly, missing results did not contribute to the risk of bias assessment, and authors of primary studies were not contacted for additional information regarding the methods or quality of their study.

### Analysis

The study selection process is summarised using a PRISMA-recommended study flow diagram (13). A summary of findings table is presented with characteristics of included studies and key outcome data relevant to our objectives. Data was managed in Excel and analysed using Stata version 18.

Due to suspected heterogeneity in how syndemic interactions were defined, investigated and reported, the first research question (syndemic patterns) was narratively synthesised; we tabulated what syndemic factors were investigated from each included study and provide a narrative synthesis on what syndemics were confirmed and in what context (reporting the statistical significance, direction and magnitude of the interaction and clusters where reported). This approach facilitated the identification of patterns, diversity in conceptual approaches, context and research gaps.

The use of a meta-analysis was considered for the second and third research questions. The suitability of conducting a meta-analysis was assessed by methodological similarities of included studies. Two pairwise random effects models (decided *a priori* due to the expectation of heterogeneity in syndemics used and participant characteristics) using the DerSimonian and Laird method were conducted (19); study standard errors were estimated for the log-transformed odds ratio, and study results were pooled on the log scale (20). Heterogeneity between the included studies was assessed with the I^2^ statistic (21). One model was used for identifying the pooled impact of syndemic count on ART adherence, and one for identifying the pooled impact of syndemic count on viral suppression.

For studies not included in the meta-analyses, a structured narrative synthesis was conducted.

## Results

### Study selection

**Figure 1:**
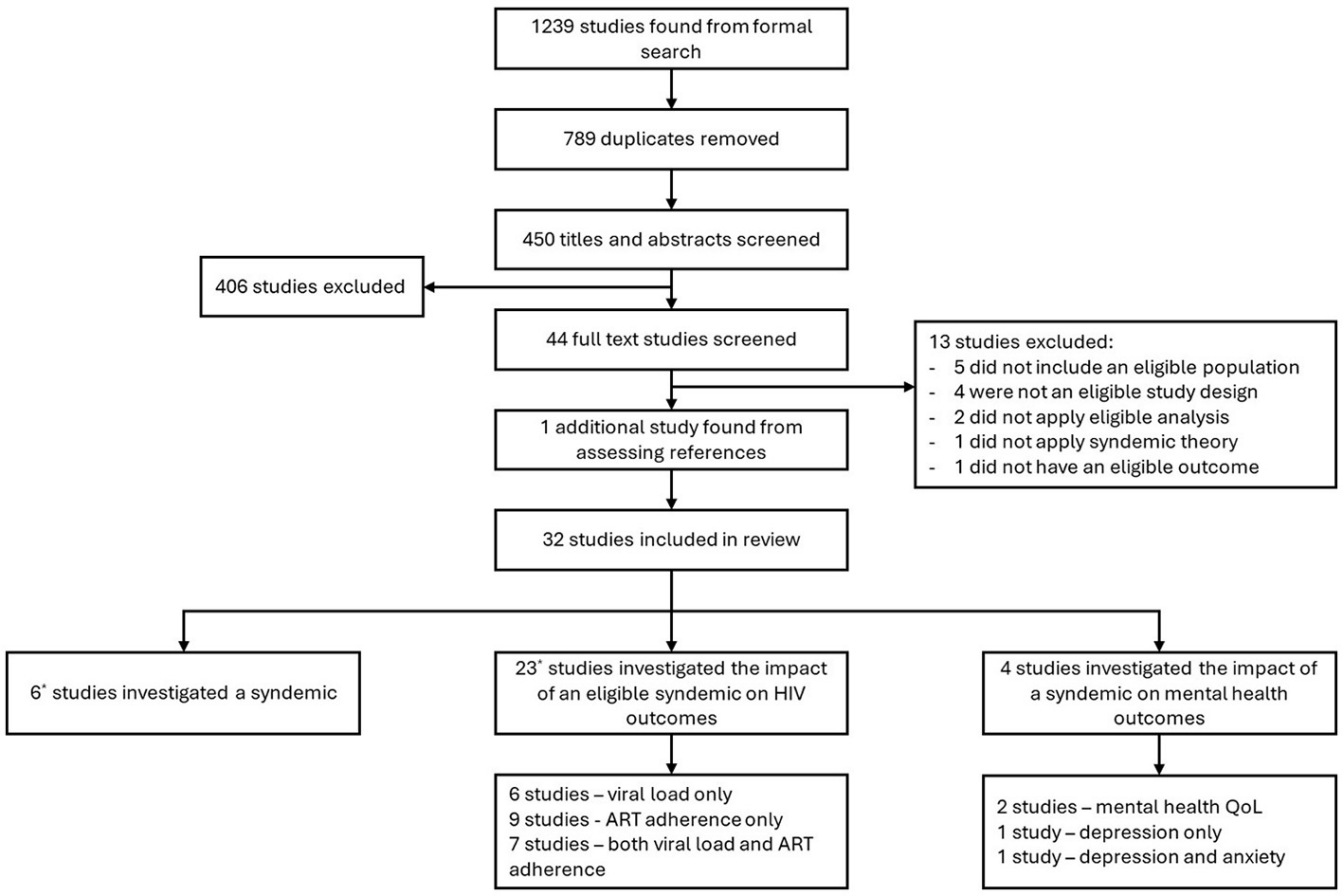
PRISMA flow Chart.

The formal search resulted in 1,239 studies, of which 789 were automatically removed within Covidence due to duplication (Figure 1). This left 450 titles and abstracts screened, followed by the screening of 44 full texts. Thirteen studies were excluded for the following reasons: five did not include PLWH only; four were not an eligible study design; two did not apply an eligible analysis; one did not apply syndemic theory; one did not have an eligible outcome. The resulting 31 studies were included. One additional eligible study was found in the informal search, resulting in 32 total studies included in this review. Five studies answered question 1 only (syndemic patterns) (22–26); one study answered both questions 1 and 2 (impact on HIV outcomes) (27); 22 studies answered question 2 only (28–48), two of which were from the same PhD thesis (31). Four studies answered question 3 (impact on mental health outcomes) (49–52).

### Study characteristics

Twenty-four of the included studies were conducted in high-income countries, 22 of which were conducted in the USA (Table 1). Five were conducted in Africa, four of which were conducted in South Africa; only three were conducted in South America, and one was conducted in Asia. Most of the included studies were cross-sectional (*n* = 20); one study was mixed-methods, for which the quantitative data were used, and the others were cohort studies (*n* = 11). The sample sizes ranged from 51 participants to 14,261; however, half had samples ranging from 200 to 400 participants (*n* = 16). More than half of the studies (*n* = 17) were conducted in 2015 or earlier, and only two had been conducted since 2020.

**Table 1:** Study characteristics.

Review outcome	Study	Setting	Study design	Sample size	Study period	Population	Key findings	NOS score
Syndemic patterns	Tsuyuki et al. (22)	USA	Cross-sectional	*n* = 481	2010–2012	Adults living with HIV and with recent substance use	Substance use, violence, and mental health create a syndemic in PLWH but patterns differed by sex and sexuality.	6
	Choi et al. (23)	South Africa	Cross-sectional	*n* = 200	2013–2014	Adult pregnant women living with HIV	Distress about pregnancy was the most overall influential factor, followed by lower income and unemployment; however, distress about pregnancy, younger age and antenatal depression were the most directly influential to other syndemic factors.	4
	Lee et al. (24)	South Africa	Cross-sectional	*n* = 194	NA	Adults living with HIV	Depression, substance use and food insecurity were the most overall and directly influential factors, correlating with each other, as well as with other syndemic factors.	4
	Gómez et al. (25)	USA	Mixed-methods	*n* = 129	2014–2019	Adult methamphetamine-using men who have sex with males living with HIV	Depression, PTSD and negative affect were the most overall influential factors (in that order); however, depression, sexual compulsivity and PTSD were the most directly influential to other syndemic factors.	3
	Wiginton et al. (26)	USA	Cross-sectional	*n* = 206	2019–2022	Young adults (15–24 years) living with HIV	Three syndemic classes were found: polydrug-socioeconomic; distress-socioeconomic; syndemic-free. Missing an ART dose in the past month and having unsuppressed viral load was most prevalent among the polydrug-socioeconomic syndemic class.	6
Syndemic patterns and HIV outcomes	Robinson et al. (27)	USA	Cross-sectional	*n* = 383	NA	Adults living with HIV with current or past drug use	Four syndemic classes were found: moderate substance use/mental illness; high mental illness; moderate substance use and mental illness/high familial conflict; high substance use/high mental illness. Having high mental illness only and having moderate substance use and mental illness plus high familial conflict was associated with suppressed viral load compared to having high substance use and high mental illness.	7
HIV outcomes	Blashill et al. (28)	USA	Cross-sectional	*n* = 333	2009–2012	Adults living with HIV and with depression symptoms	Compared to having no syndemics, having 3–4 or 5+ syndemics were associated with non-adherence. Having 1–2 syndemics compared to having no syndemics was not associated with non-adherence.	5
	Biello et al. (29)	Argentina, Bolivia, Brazil, Chile, Colombia, Costa Rica, Ecuador, El Salvador, Guatemala Honduras, Mexico, Nicaragua, Panama, Paraguay, Peru, Uruguay, Venezuela	Cross-sectional	*n* = 2020	2012	Adult men who have sex with men living with HIV	Every increase in the number of syndemic factors was associated with a 14% reduction in odds of being 100% adherent to ART.	2
	McMahon et al. (30)	USA	Cross-sectional	*n* = 317	2011–2012	Adult heterosexual men living with HIV	A 1 unit increase on the stigma scale was associated, through heightened anxiety, with a 27% lower probability of optimal adherence. A 1 unit increase on the social support scale, and associated decrease in drug dependence, resulted in a 7% higher probability of optimal adherence.	6
	Myers (31)	USA	Cohort	*n* = 134	2009–2014	Adults living with HIV	For every increase in the number of syndemic factors, there was a 14% reduction in 4-day adherence and 13% reduction in 3-month adherence, but neither were significant.	4
	Holloway et al. (32)	USA	Cross-sectional	*n* = 98	2018–2019	Adult Black men who have sex with men living with HIV	For every increase in structural syndemics, the mean number of missed ART doses increases by a factor of 1.63. The increase in psychosocial syndemics did not have a significant impact on adherence.	2
	Lee et al. (33)	South Africa	Cohort	*n* = 194	2019–2021	Adults living with HIV	The only significant impact on ART adherence was the interaction between depression and food insecurity; among individuals with 1 standard deviation below average food insecurity, increasing severity of depressive symptoms was associated with decreased adherence.	5
	Rutledge et al. (34)	USA	Cohort	*n* = 302	2015–2018	Older (40+ years) African American men living with HIV	For every increase in the number of syndemics, ART adherence reduced by a factor of 0.018.	4
	Rodríguez-Díaz et al. (35)	Brazil, Thailand, Zambia	Cohort	*n* = 692	2011–2013	Adults living with HIV	Compared to having no syndemic factor, having 2 or 3+ significantly reduced ART adherence. Having 1 syndemic did not have a significant impact.	6
	Watson (36)	USA	Cohort	*n* = 969	1994–1995 2001–2002 2011–2012 2013–2015	Middle-aged (30–55 years) women living with HIV	A higher number of any syndemic and a higher number of neighbourhood-level structural syndemics was associated with higher odds of non-adherence. Compared to having no syndemics, having syndemics in 2 or all 3 of the specified syndemic groups (psychosocial, participant-level and neighbourhood-level) had higher odds of non-adherence.	6
	Wawrzyniak et al. (37)	USA	Cross-sectional	*n* = 444	2013–2014	Adults living with HIV	Compared to having no individual-level syndemic, having 2 or 3+ significantly reduced odds of being virally suppressed. Having 1 syndemic did not have a significant impact, neither did any number of systemic syndemic.	8
	Friedman et al. (38)	USA	Cohort	*n* = 712	2002–2009	Adult gay or bisexual men living with HIV	Increasing number of syndemics had a negative impact on viral suppression and social support had a moderating effect.	8
	Yellin et al. (39)	USA	Cross-sectional	*n* = 106	2013–2015	Adults living with HIV	Compared to having no syndemic factor, having 1, 2 or 3 significantly reduced odds of being virally suppressed.	5
	Myers 2019 (31)	USA	Cohort	*n* = 51	2009–2014	Adults living with HIV	The number of syndemic factors did not significantly impact the odds of being virally suppressed.	5
	Jones et al. (40)	USA	Cross-sectional	*n* = 131	2010–2011	Adult women living with HIV	After adjusting for age, the number of syndemic factors were not significantly associated with viral load.	5
	Bhardwaj et al. (41)	South Africa	Cross-sectional	*n* = 1384	2018–2020	Adult female sex workers living with HIV	Compared to having no depression or SAVA syndemic, having just depression, having SAVA without depression, and the interaction between depression and SAVA had a significant impact on being virally suppressed.	7
	Friedman et al. (42)	USA	Cohort	*n* = 766	2003–2009	Adult men who have sex with men living with HIV	Compared to having no syndemics, 1 syndemic was associated with reduced adherence and higher viral load as was 2 syndemics compared to 1 and 3 syndemics compared to 2.	6
	Kuhns et al. (43)	USA	Cross-sectional	*n* = 212	2011–2014	Young adults (16–29 years) living with HIV	Compared to having no syndemic factors, having 3 or 4 had significantly impacted adherence; having 1, 2 or 5 reduced adherence but were not significant. Having 4 syndemic factors significantly reduced the odds of being virally suppressed; having 1, 2, 3 or 5 also reduced the odds but was not significant. Linear dose for number of syndemic factors related to both ART adherence and viral suppression was significant.	5
	Harkness et al. (44)	USA	Cohort	*n* = 390	2004–2008	Adult men who have sex with males living with HIV	Number of syndemics was a significant longitudinal predictor of non-adherence and detectable viral load, with each additional syndemic associated with a 0.13 increase in non-adherence and 1.27 greater odds of detectable viral load.	5
	Glynn et al. (45)	USA	Cross-sectional	*n* = 800	2017–2018	Adults living with HIV	Increase in the number of syndemics significantly reduced the odds of adherence and being virally suppressed.	6
	Zepf et al. (46)	USA	Cross-sectional	*n* = 281	2012–2016	Older (50+ years) gay or bisexual men living with HIV	ART adherence differed between those with 1 to no syndemic factor and those with 2 or more; the former had better adherence. There was no difference with viral load.	5
	Satyanarayana et al. (47)	USA	Cohort	*n* = 14261	2007–2017	Adults living with HIV	Increase in the number of syndemics significantly reduced the odds of adherence and of being virally suppressed.	6
	Shayegi-Nik et al. (48)	Canada	Cohort	*n* = 12076	2001–2019	Adults living with HIV	Compared to having no syndemic, having substance use disorder and a mood/anxiety disorder reduced odds for being adherent and virally suppressed.	4
Mental health outcomes	Mesías-Gazmuri et al. (49)	Spain	Cross-sectional	*n* = 861	2019–2020	Adults living with HIV	An interaction between social role and cognitive function resulted in worse mental health-related quality of life.	3
	Gomes et al. (50)	Brazil	Cross-sectional	*n* = 1530	2014–2017	Adults living with HIV	Depression, polysubstance use, intimate partner violence and the presence of 2 or more syndemic conditions were associated with worse mental health-related quality of life.	6
	Thurston et al. (51)	USA	Cross-sectional	*n* = 55	2014–2015	Adult mothers living with HIV	SAVA increased depressive symptoms but only when resilience was low.	4
	Maclin et al. (52)	Dominican Republic	Cross-sectional	*n* = 311	2018–2019	Adult female sex workers living with HIV	Sex work-related police harassment syndemic cluster among cisgender females and sex work-related violence and harassment syndemic cluster among transgender females had greater odds of having anxiety and depression.	4

SAVA, substance use, violence and AIDS.

Less than half (*n* = 12) included adult PLWH without restriction on sex, sexuality, or a particular exposure (e.g. substance use). Seven studies included men who have sex with men (MSM); six included women only, two of which were specific to female sex workers; three included PLWH with past or current substance use; two included younger adult PLWH and; one study each included heterosexual adult PLWH, older adult Black men PLWH, and PLWH with depressive symptoms. These are not mutually exclusive.

A range of syndemic factors were investigated across the 32 studies (Table 2). For research questions 1 and 2: depression was the most commonly eligible mental health condition included (*n* = 24); 10 studies included anxiety, seven included PTSD; two included distress; five used a composite measure and; one study included schizophrenia and bipolar disorder. Across all 32 studies, 18 included alcohol use as part of the syndemic, and 27 included drug use. Experience of violence, either currently or in the past, was included in 14 studies, and five specifically included childhood abuse. Several factors that were investigated related to socioeconomic status included unstable housing (*n* = 8), poverty/financial insecurity/low income (*n* = 6), food insecurity (*n* = 5) and low education attainment (*n* = 5). Stigma was included in eight studies, whereas four studies used barriers to HIV care. Only a few studies included social-related factors such as social support (*n* = 3) and caregiving responsibilities (*n* = 2), sexual-related factors such as sexual compulsivity (*n* = 3) and condomless sex (*n* = 2), criminal activity (*n* = 4) and physical health-related factors (*n* = 2). How each study defined and measured syndemic factors is described in the Supplementary material.

**Table 2:** Syndemics investigated across included studies.

Study	Depression	Anxiety	Post-traumatic stress disorder	Bipolar disorder	Schizophrenia	Negative affect	Distress	Quality of life	Composite mental health condition	Suicidal ideation	Cognitive function	Alcohol use	Drug use	Cigarette use	Childhood abuse	Past or current violence	Police harassment	Unstable housing	Poverty / financial insecurity / income	Food insecurity	Low education	Local socioeconomic environment	Unemployment	Stigma	Structural barriers to HIV care	Quality of HIV care	Dysfunctional relationships	Social support	Caregiving responsibilities	Instability	Sexual compulsivity	Sexual satisfaction	Condomless anal sex	Incarceration	Accused or charged with a crime	Obesity	Disability
Tsuyuki et al. (22)	✓	✓					✓						✓			✓																					
Choi et al. (23)	✓						✓									✓			✓		✓		✓	✓				✓	✓								
Lee et al. (24)	✓	✓	✓									✓	✓			✓		✓	✓	✓					✓												
Gómez et al. (25)	✓		✓			✓			✓			✓	✓					✓									✓				✓			✓			✓
Robinson et al. (27)	✓	✓	✓	✓	✓								✓														✓										
Wiginton et al. (26)	✓	✓										✓	✓					✓		✓				✓											✓		
Blashill et al. (28)									✓						✓	✓																					
Friedman et al. (38)	✓												✓																				✓				
Wawrzyniak et al. (37)	✓							✓					✓							✓					✓	✓		✓	✓	✓							
Biello et al. (29)	✓									✓		✓	✓		✓	✓															✓						
Kuhns et al. (43)	✓	✓										✓	✓											✓													
Friedman et al. (42)	✓												✓																				✓				
Harkness et al. (44)	✓	✓	✓									✓	✓		✓																						
Yellin et al. (39)									✓			✓	✓																								
Glynn et al. (45)	✓	✓										✓	✓			✓		✓			✓			✓													
McMahon et al. (30)		✓											✓											✓				✓									
Myers (31)	✓											✓	✓								✓																
Myers (31)	✓											✓	✓								✓																
Jones et al. (40)	✓													✓							✓															✓	
Zepf et al. (46)	✓		✓									✓	✓			✓																					
Holloway et al. (32)	✓											✓	✓		✓	✓		✓	✓																✓		
Satyanarayana et al. (47)	✓	✓										✓	✓																								
Watson (36)	✓											✓	✓		✓	✓		✓	✓	✓		✓		✓	✓									✓			
Bhardwaj et al. (41)	✓											✓	✓			✓																					
Lee et al. (33)	✓	✓	✓									✓	✓			✓		✓	✓	✓					✓												
Rutledge et al. (34)	✓		✓									✓	✓					✓																			
Shayegi-Nik et al. (48)									✓				✓																								
Rodríguez-Díaz et al. (35)	✓											✓	✓											✓													
Mesías-Gazmuri et al. (49)	✓										✓			✓					✓					✓			✓					✓					
Thurston et al. (51)													✓			✓																					
Gomes et al. (50)	✓											✓	✓			✓															✓						
Maclin et al. (52)																✓	✓																				

### Quality of included studies

The average number of stars given across the 32 studies was 5.03. This ranged from one star (one study) to 8 stars (two studies). The full breakdown of stars given for each NOS question for each study is available in the Supplementary materials.

### Investigation of a syndemic (Question 1)

Three studies used a network analysis to confirm syndemic patterns, reporting bidirectional associations between factors. Choi et al. (23) investigated patterns among nine syndemic factors and found that among 200 adult pregnant women in South Africa, distress about pregnancy was the most overall influential factor (most central node) and positively associated with unintended pregnancy (*b* = 0.58), antenatal depression (*b* = 0.33) and HIV stigma (*b* = 0.27). Distress about pregnancy was negatively associated with younger age (*b* = −0.38) and lower income (*b* = −0.21). Lower income was the second most influential factor and was positively associated with unemployment (*b* = 1.11) and antenatal depression (*b* = 0.12). Authors then conducted a community detection analysis, which identified three clusters: socioeconomic risk, psychosocial distress and miscellaneous social risks.

Lee et al. (24) investigated patterns between 10 syndemic factors and found that among 194 adults in South Africa, depression was the most influential factor in the network analysis. Depression was significantly associated with PTSD (*b* = 0.33), social anxiety (*b* = 0.19) and food insecurity (*b* = 0.18). Substance use, the next most influential factor, was only significantly associated with alcohol use (*b* = 0.30). Food insecurity (the third most influential factor) was significantly associated with poverty (*b* = 0.30), and PTSD (the fourth most influential factor) was significantly associated with social anxiety (*b* = 0.20) and intimate partner violence (b=0.22). The authors applied the spinglass algorithm to the network and identified two clusters: mood- and violence-related syndemic problems and substance use and structural barriers to care cluster.

Gomez et al. (25) investigated patterns between 11 syndemic factors and also reported depression as the most influential factor from their network analysis among 129 methamphetamine-using MSM in the USA. Depression was significantly associated with PTSD (*b* = 0.37) and negative affect (*b* = 0.26). The remaining associations found were not significant; however, the authors report two main clusters from visual examination of the network analysis: a psychosocial-affective cluster and a structural cluster.

Tsuyuki et al. (22) conducted a structural equation modelling (SEM) confirmatory factor analysis (CFA) with data from 481 adults with recent substance use in the USA, stratified by sex and sexuality. Within a syndemic of violence, depression, anxiety, substance use and stress, anxiety and depression were the strongest contributors among heterosexual men (*b* = 1.53 and 1.33, respectively), MSM (*b* = 1.50 and *b* = 1.17, respectively) and heterosexual women (*b* = 1.23 and *b* = 0.98, respectively). Stress was the weakest contributor among all three groups (*b* < 0.50), and substance use varied between heterosexual men (*b* = 0.46), MSM (*b* = 0.61) and heterosexual women (*b* = 0.78).

Robinson et al. (27) and Wiginton et al. (26) both conducted a latent class analysis. The former identified four clusters among 383 adults with current or past drug use in the USA. Cluster 1 was moderate substance use and mental illness, which contained 43% of the sample and comprised a moderate proportion of people who used cocaine (33%), heroin (21%) and had depressive symptoms (30%). Cluster 2 was high mental illness and included 25% of the sample with a high burden of depression (93%), anxiety/PTSD (43%) and bipolar disorder (69%). Cluster 3 was moderate substance use and mental illness and high familial conflict non-negotiation; 23% of the sample was in this cluster and 41% used cocaine, 24% used heroin, 21% used marijuana, 65% had depression, 94% said family rarely or never talks about problems and 95% said family rarely or never problem solves together. The final cluster was high substance use and mental illness, representing 9% of the sample; 100% used cocaine, 48% used marijuana, 71% used heroin, 95% had depression, 61% had anxiety/PTSD and 76% had bipolar disorder.

Among a sample of 206 young adults in the USA, Wiginton et al. (26) identified three clusters. The first cluster, polydrug-socioeconomic syndemic, comprised 14% of the sample with high use of marijuana (97%) and alcohol (71%), and moderate prevalence of legal involvement (65%) and food insecurity (~62%; exact figures not provided). Cluster 2 was distress-socioeconomic syndemic comprising 17% of the sample: 96% had depression, 95% had anxiety, 60% used marijuana, around 47% had food insecurity, and around 41% had legal involvement (exact figures not reported). The majority of the sample (69%) was in the syndemic-free cluster, which had a low burden of syndemic factors other than a small proportion for marijuana use (36%). Having missed an ART dose in the previous month was most prevalent within the polydrug-socioeconomic cluster (89%), followed by the distress-socioeconomic cluster (71%), followed by the syndemic-free cluster (59%)

### Impact on HIV outcomes (question 2)

#### ART adherence

Thirteen studies investigated the impact of increasing syndemic factors on ART adherence. Of these, twelve found that with an increasing number of syndemics, ART adherence was significantly reduced; however, one of these studies (32) found this only for structural syndemics (i.e. poverty, criminal justice involvement and housing instability) and not for psychosocial syndemics (i.e. alcohol-related problems, drug-related problems, intimate partner violence, depression and childhood sexual abuse) as described by the study authors. Myers (31) also reported a negative relationship between syndemic count and ART adherence, but the results were not significant.

**Figure 2:**
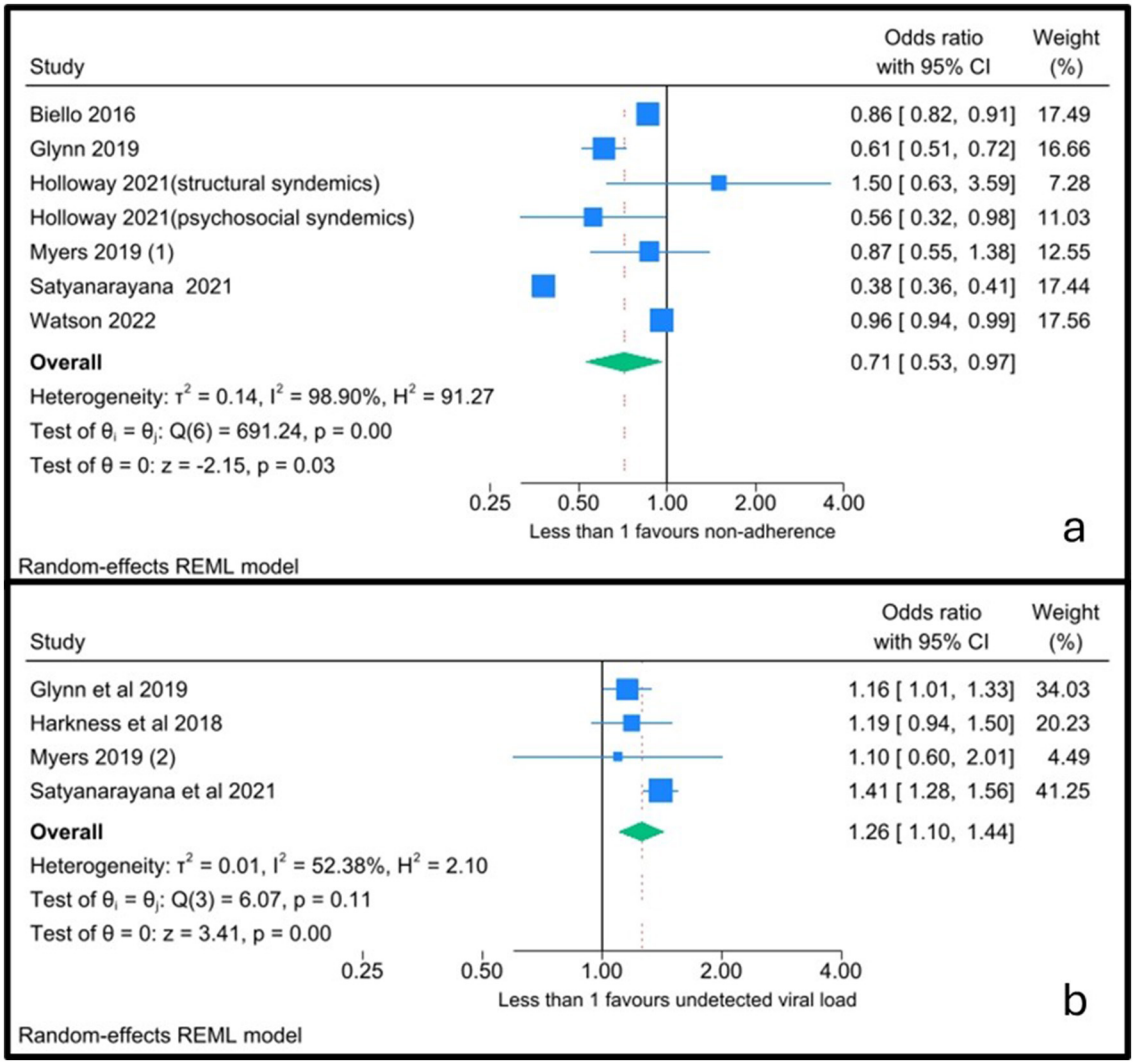
Pooled analysis for ART adherence **(A)** and viral suppression **(B)**.

Six studies provided enough data to conduct a meta-analysis, of which one study provided two separate analyses (32): one for the impact on structural syndemics and one for the impact on psychosocial syndemics. Four studies reported odds ratios below 1.0, indicating a consistent direction of effect whereby increasing syndemic burden was associated with poorer ART adherence (Figure 2a). The magnitude of effects varied across studies, with three studies having wide confidence intervals and therefore contributed less to the pooled estimate. The pooled odds ratio was significant, indicating that for every increase in the number of syndemic factors, adherence is reduced by 29% (pooled OR 0.71, 95% CI 0.53–0.97). However, heterogeneity was high (*I*^2^ = 98.90%), and it remained high in a *post-hoc* analysis with only studies that included adult PLWH with no population-specific eligibility criteria (*n* = 3; *I*^2^ = 94.95%; Supplementary material).

The three remaining studies used a different approach to report the impact of syndemics on ART adherence. Zeph et al. (46) reported a significant difference between MSM with 0–1 syndemics and MSM with 2+ syndemics and the proportion of MSM reporting very poor, poor, fair, good, very good or excellent adherence, with a higher proportion reporting excellent adherence in the 0–1 syndemic group (73 vs. 44%). McMahon et al. (30) found that adherence was reduced with every unit increase in the stigma scale when anxiety was present (significant interaction). Authors also reported an increase in adherence with every unit increase in social support when drug dependence was decreased. Lee et al. (33) found that when individuals were 1 standard deviation below average for food insecurity, an increase in depression severity negatively impacted ART adherence.

#### Viral suppression

Of the 11 studies that investigated the impact of the number of syndemics on the odds of being virally suppressed, ten found that an increase in syndemics reduced the odds of being virally suppressed. One of these studies (37) found this for individual-level syndemics (i.e. depression, drug-use, quality of life, food insecurity, instability, adherence, social support instability and caregiving responsibilities) but not for systemic syndemics (i.e. various structural barriers to HIV care such as transportation, language, appointment difficulty etc.). Another study (38) found that social support had a moderating effect on the relationship between syndemic count and viral suppression. Myers (31) reported a negative relationship between syndemic count and viral suppression, but this was not significant.

Only four studies provided the appropriate data for a meta-analysis, two of which reported odds ratios above 1.0, indicating higher odds of detectable viral load with increasing syndemic burden (Figure 2b). One study contributed minimal weight to the pooled estimate due to very wide confidence intervals, reflecting substantial imprecision. Overall, the pooled estimate suggests that each additional syndemic factor is associated with a 26% increased odds of having detectable viral load (pooled OR 1.26; 95% CI 1.10, 1.44). Between-study heterogeneity was moderate (*I*^2^ = 52.38%), indicating some variability in effect sizes but reasonable consistency in the direction of effect.

Zeph et al. (46) reported a slightly higher proportion of MSM with suppressed viral load in the 0–1 syndemic group (84%) compared to the 2+ syndemic group (81%), but this was not significant. Bhardwaj et al. (41) investigated the impact of different combinations between SAVA (defined as substance abuse, violence and AIDS) and depression on viral suppression; they found that all combinations resulted in reduced prevalence of viral suppression in comparison to those with no depression, substance use or violence.

### Impact on mental health outcomes (question 3)

Mesias-Gazmuri et al. (49) found that an interaction between social role and cognitive function had a negative impact on mental health-related quality of life among adult PLWH in Spain, measured using the SF-12 health questionnaire (53). Gomes et al. (50) found that having 2 or more syndemic factors also significantly reduced mental health-related quality of life among adult PLWH in Brazil, measured using the WHOQOL-HIV-BREF (54). Thurston et al. (51) reported an increase in depressive symptoms with the presence of substance use and violence among adult mothers living with HIV in the USA. Maclin et al. (52) reported that specific clusters of violence-related syndemics had an impact on mental health among different groups of adult female sex workers in the Dominican Republic: sex work-related police harassment syndemic cluster among cisgender females and sex work-related violence and harassment syndemic cluster among transgender females had greater odds of having anxiety and depression.

## Discussion

**Figure 3:**
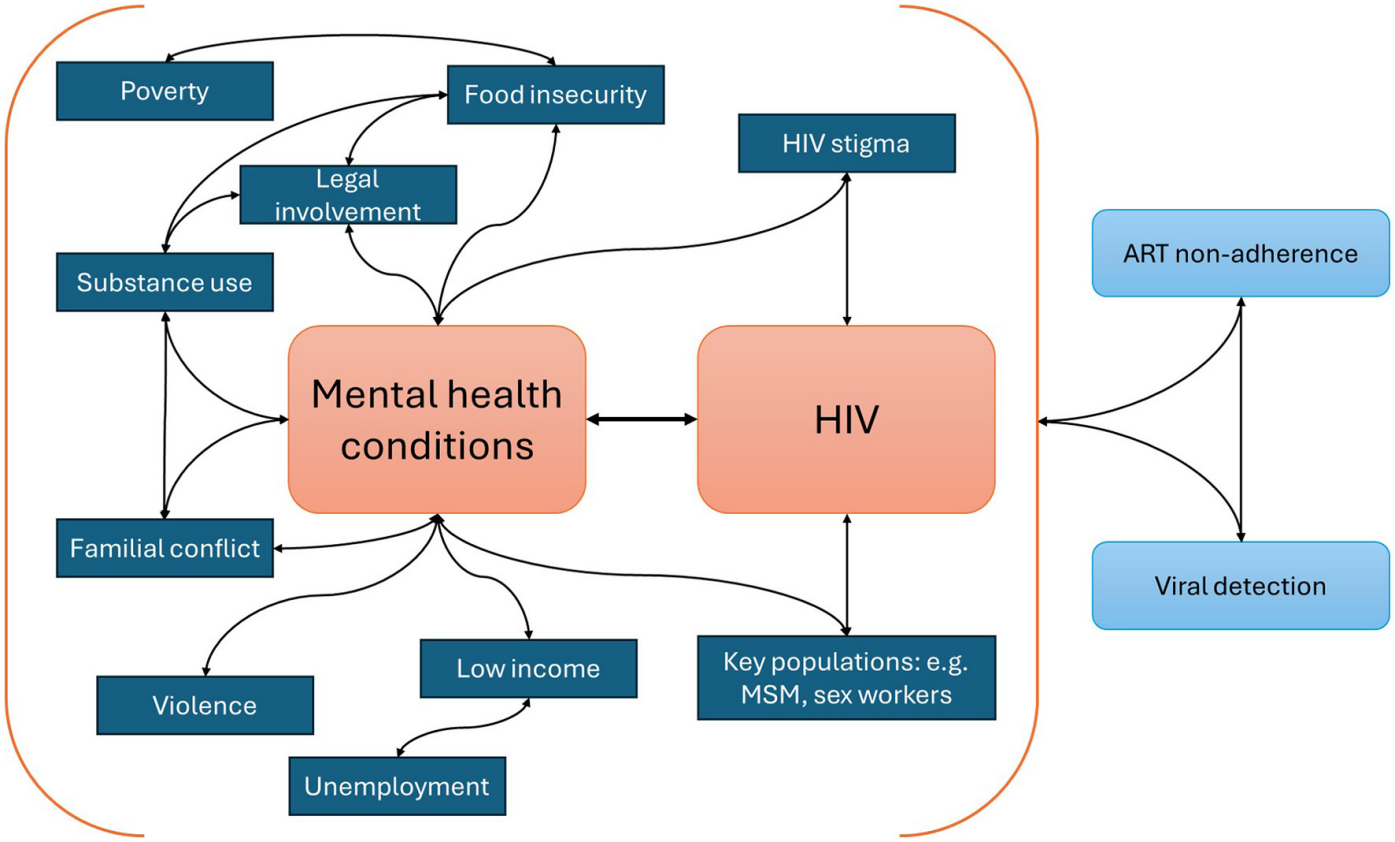
Mental health and HIV syndemic.

This review synthesised evidence from 32 studies to better understand the interaction of mental health and HIV through a syndemic lens. Depression was consistently a large contributor to syndemic patterns across a diverse set of studies that investigated patterns and interactions of syndemic factors among PLWH. Depression was found to interact with other mental health conditions and often with psychosocial factors such as stigma, food insecurity, drug use and violence. We found that ART adherence decreased and viral load increased as the number of syndemics increased; this was irrespective of the diverse factors investigated alongside mental health conditions. These findings, summarised visually in Figure 3, reinforce syndemic theory and highlight the need for holistic interventions to address co-occurring mental health, substance use and psychosocial disparities among PLWH.

Our results suggest that depression may function as a key syndemic amplifier rather than simply a co-occurring condition. Depression and other mental health conditions are associated with syndemic factors among the general population, such as substance use, exposure to violence and unemployment (55), as well as PLWH, as our review found. However, both depression and HIV disproportionately affect disadvantaged and marginalised populations such as MSM and female sex workers, who in many settings face legal barriers and pervasive discrimination (56–59). Depression and its associated syndemic factors may therefore predate HIV diagnosis and be exacerbated following diagnosis through HIV-related stigma and the psychological burden of living with a chronic condition (e.g. lifelong and often daily ART, regular healthcare appointments), particularly among populations who are otherwise physically well. Socially and structurally, depression among PLWH is strongly shaped by stigma, violence, poverty, food insecurity, and social isolation. Within a syndemic context, depression may both arise from and intensify co-occurring psychosocial stressors, generating reinforcing feedback loops that heighten vulnerability to poor HIV outcomes (ART adherence and viral suppression) (60, 61). Important to note, ART exposure and HIV viral presence may also contribute to the aetiology and persistence of depressive symptoms (e.g. central nervous system dysregulation) (4–6); however, this was not within the scope of this review.

We found only one study conducted in sub-Saharan Africa outside South Africa, highlighting a major gap in research regarding the synergies between, and the impact of, mental health and other environmental, social, political and economic factors among PLWH in this region. 65 percent of PLWH live in sub-Saharan Africa, where PLWH experience disproportionate and differentiating exposure to intersecting social and structural vulnerabilities related to persistent poverty, violence, food insecurity, unemployment, gender inequality, conflict and displacement, stigma and discrimination, unstable housing, dysfunctional families and lack of social support (1, 62). Thus, the limited number of studies likely reflects several structural and methodological barriers rather than an absence of syndemics. For instance, mental health conditions remain underdiagnosed and under-measured in many African settings due to shortages of trained personnel, limited use of validated screening tools, and/or competing clinical priorities within overstretched health systems (63). Additionally, local conceptualisations of mental distress, gender norms, and HIV-related stigma may further influence both the expression and reporting of syndemic conditions, complicating direct comparison with studies from high-income countries (64). As a result, the current evidence base, which is heavily weighted toward high-income countries, may have limited external validity for sub-Saharan Africa, highlighting the need for contextually grounded syndemics research that reflects local social, cultural, and health system realities.

Several additional gaps in the literature were highlighted from our review. For instance, our synthesis reveals that the mental health dimensions of syndemics have been examined unevenly. Depression was overwhelmingly the most studied condition, whereas anxiety, PTSD, and distress were included far less frequently, and schizophrenia and bipolar disorder were nearly absent from the literature. Given that these conditions are uniquely associated with various psychosocial factors (65–67) and are more prevalent among PLWH compared to people without HIV (2), their underrepresentation represents a critical research gap. A further gap identified in this review concerns the limited evidence on how syndemics influence mental health outcomes among PLWH. Only four studies examined this relationship. This scarcity of research restricts understanding of how syndemic conditions may exacerbate psychiatric morbidity among PLWH to help inform initiatives for prevention.

This is the first review to systematically synthesise existing evidence and conduct a meta-analysis for understanding the synergies of mental health and HIV. However, there are some limitations to mention. The studies included in the meta-analyses had substantial heterogeneity, limiting the precision of the effect estimates. However, through individual studies, consistent findings indicate that mental health-related syndemics negatively impact HIV outcomes irrespective of population and settings. Syndemics measured varied widely across the included studies, proving difficult to synthesise; however, depression consistently was the most influential factor within syndemic clusters.

The findings from this systematic review and meta-analysis demonstrate that mental health conditions, particularly depression, are a leading contributor to syndemic patterns among PLWH, and an increasing number of syndemic factors is associated with poorer ART adherence and detectable viral load. Syndemics among PLWH represent a conceptual and empirical bridge between mental health and HIV, supporting calls to investigate the effectiveness of integrating mental health care with chronic disease management, routine screening and evidence-based treatment. Our review also highlights that interventions to address mental health-related syndemics must be holistic, moving beyond condition-specific care to incorporate structural and psychosocial determinants of health. However, more syndemic research is desperately needed in low- and middle-income countries where the burden of HIV, mental health conditions and psychosocial factors is high and differs from high-income countries.
