# Supplementary material for: Syndemics and clinical impact of HIV and mental health conditions among people living with HIV: a systematic review and meta-analysis
Source: Front Public Health. 2026 Apr 10;14:1778334. doi: 10.3389/fpubh.2026.1778334 (PMC13137443; doi:10.3389/fpubh.2026.1778334)
Supplement: Supplementary file 1 [file Table_1.docx]

**Search strategies**

MEDLINE (ALL 1946 to June 10, 2025)

|  | Search terms | Number of hits* |
| --- | --- | --- |
| 1 | exp HIV Infections/ or exp HIV/ or HIV.ti,ab. or "human immunodeficiency virus".ti,ab. | 471980 |
| 2 | exp Syndemic/ or syndemic*.ti,ab. | 1461 |
| 3 | exp Depression/ or depress*.ti,ab. | 651809 |
| 4 | exp Generalized Anxiety Disorder/ or exp Anxiety/ or exp Anxiety Disorders/ or anxi*.ti,ab. | 401636 |
| 5 | exp Schizophrenia/ or schizophren*.ti,ab. | 171205 |
| 6 | exp Bipolar Disorder/ or bipolar.ti,ab. | 97141 |
| 7 | exp Stress Disorders, Post-Traumatic/ or exp Stress, Psychological/ or PTSD.ti,ab. or stress.ti,ab. or distress.ti,ab. | 1318881 |
| 8 | 3 or 4 or 5 or 6 or 7 | 2224447 |
| 9 | 1 and 2 and 8 | 256 |

* Search was conducted on 11 June 2025

CINHL Plus

|  | Search terms | Number of hits* |
| --- | --- | --- |
| 1 | (MH "HIV Infections+") OR (MH "HIV+") OR (MH "Acquired Immunodeficiency Syndrome") OR HIV OR "human immunodeficiency virus" | 144260 |
| 2 | XB syndemic OR XB syndemics OR XB syndemic theory | 756 |
| 3 | (MH "Depression+") OR depression OR depressive | 235944 |
| 4 | (MH "Anxiety Disorders+") OR anxiety OR anxious | 179288 |
| 5 | (MH "Schizophrenia+") OR schizophrenia OR schizophrenic | 40314 |
| 6 | (MH "Bipolar Disorder+") OR "bipolar disorder" OR bipolar | 22778 |
| 7 | (MH "Post Traumatic Stress Disorder+") OR (MH "Emotional Distress") OR PTSD OR stress OR distress | 336629 |
| 9 | 3 or 4 or 5 or 6 or 7 | 631943 |
| 10 | 1 and 2 and 8 | 176 |

* Search was conducted on 13 June 2025

Embase via OVID (1974 to 2025 June 10)

|  | Search terms | Number of hits* |
| --- | --- | --- |
| 1 | exp Human immunodeficiency virus/ or HIV.ti,ab. or "human immunodeficiency virus".ti,ab. | 549521 |
| 2 | exp syndemic/ or syndemic*.ti,ab. | 1701 |
| 3 | exp major depression/ or exp chronic depression/ or exp depression/ or exp minor depression/ or depress*.ti,ab. | 1099754 |
| 4 | exp anxiety/ or exp anxiety disorder/ or exp generalized anxiety disorder/ or anxi*.ti,ab. | 783346 |
| 5 | exp schizophrenia/ or schizophren*.ti,ab. | 255798 |
| 6 | exp bipolar I disorder/ or exp bipolar disorder/ or exp bipolar II disorder/ or bipolar.ti,ab. | 153003 |
| 7 | exp posttraumatic stress disorder/ or PTSD.ti,ab. or stress.ti,ab. or distress.ti,ab. | 1609382 |
| 8 | 3 or 4 or 5 or 6 or 7 | 3021767 |
| 9 | 1 and 2 and 8 | 325 |

* Search was conducted on 11 June 2025

APA PsycInfo (1967 to June 2025 Week 1)

|  | Search terms | Number of hits* |
| --- | --- | --- |
| 1 | exp HIV/ or HIV.ti,ab. or "human immunodeficiency virus".ti,ab. | 66336 |
| 2 | syndemic*.ti,ab. | 666 |
| 3 | exp Major Depression/ or depress*.ti,ab. | 385983 |
| 4 | exp Anxiety Disorders/ or exp Anxiety/ or exp Generalised Anxiety Disorder/ or anxi*.ti,ab. | 284510 |
| 5 | exp Schizophrenia/ or schizophren*.ti,ab. | 132486 |
| 6 | exp Bipolar Disorder/ or exp Bipolar I Disorder/ or exp Bipolar II Disorder/ or bipolar.ti,ab. | 52669 |
| 7 | exp Posttraumatic Stress Disorder/ or PTSD.ti,ab. or stress.ti,ab. or distress.ti,ab. | 346283 |
| 8 | 3 or 4 or 5 or 6 or 7 | 908895 |
| 9 | 1 and 2 and 8 | 195 |

* Search was conducted on 11 June 2025

Scopus

|  | Search terms | Number of hits* |
| --- | --- | --- |
| 1 | TITLE-ABS(HIV OR "human immunodeficiency virus") AND TITLE-ABS(syndemic*) AND TITLE-ABS(depress* OR anxi* OR schizophren* OR bipolar OR PTSD OR stress OR distress) | 244 |

* Search was conducted on 13 June 2025

ProQuest Dissertations & Theses Global

|  | Search terms | Number of hits* |
| --- | --- | --- |
| 1 | ABSTRACT,TITLE(HIV OR human immunodeficiency virus) AND ABSTRACT,TITLE(syndemic*) AND ABSTRACT,TITLE(depress* OR anxi* OR schizophren* OR bipolar OR PTSD OR stress OR distress) | 539 |
| 2 | Restriction applied=Dissertations & Theses | 43 |

* Search was conducted on 13 June 2025
