# Supplementary material for: Syndemics and clinical impact of HIV and mental health conditions among people living with HIV: a systematic review and meta-analysis
Source: Front Public Health. 2026 Apr 10;14:1778334. doi: 10.3389/fpubh.2026.1778334 (PMC13137443; doi:10.3389/fpubh.2026.1778334)
Supplement: Supplementary file 2 [file Table_2.docx]

Table 1. NOS results for cross-sectional studies

| Study | Selection | | Exposure(s) and outcome(s) | | Confounding factors | | Total stars |
| --- | --- | --- | --- | --- | --- | --- | --- |
|  | Representativeness of the study sample | Sample size | Assessment of exposure(s) | Assessment of outcome(s) | Adjustment of confounders | Assessment of confounders |  |
| Tsuyuki et al 2017 | * |  | * | * | ** | * | 6 |
| Choi et al 2019 |  |  | * | * | * | * | 4 |
| Lee et al 2023 |  |  | * | * | * | * | 4 |
| Gómez et al 2024 |  |  |  | * | ** |  | 3 |
| Wiginton et al 2024 | * |  |  | ** | ** | * | 6 |
| Robinson et al 2016 |  | * | ** | * | ** | * | 7 |
| Blashill et al 2015 |  |  | * | ** | * | * | 5 |
| Biello et al 2016 |  |  |  |  | * | * | 2 |
| McMahon et al 2019 | * | * | * | * | * | * | 6 |
| Holloway et al 2021 |  |  |  | * | * |  | 2 |
| Wawrzyniak et al 2015 | * | * | * | ** | ** | * | 8 |
| Yellin et al 2018 |  |  |  | ** | ** | * | 5 |
| Jones et al 2020 |  |  |  | ** | ** | * | 5 |
| Bhardwaj et al 2023 | * | * | * | * | ** | * | 7 |
| Kuhns et al 2016 | * | * |  |  | ** | * | 5 |
| Glynn et al 2019 | * |  | * | * | ** | * | 6 |
| Zepf et al 2020 |  |  |  | ** | ** | * | 5 |
| Mesías‑Gazmuri et al 2023 |  | * |  |  | * | * | 3 |
| Gomes et al 2023 |  | * | * | * | ** | * | 6 |
| Thurston et al 2018 |  |  | * | * | * | * | 4 |
| Maclin et al 2023 |  | * | * | ** |  |  | 4 |

Table 2. NOS results for cohort studies

| Study | Selection | | | | Comparability | Outcome | | | Total stars |
| --- | --- | --- | --- | --- | --- | --- | --- | --- | --- |
|  | Representativeness of the exposed group | Selection of non-exposed group | Ascertainment of exposure(s) | Outcome not present at start of study | Comparability of exposed and non-exposed group | Assessment of outcome | Length of follow up | Adequacy of follow up |  |
| Myers 2019 (1) |  | * |  | * | * |  | * |  | 4 |
| Lee et al 2024 |  | * |  | * | * | * | * |  | 5 |
| Rutledge et al 2024 |  | * |  | * | * |  | * |  | 4 |
| Rodriguez-Diaz et al 2025 |  | * | * | * | * |  | * | * | 6 |
| Watson 2022 |  | * | * | * | * |  | * | * | 6 |
| Friedman et al 2017 | * | * | * | * | * | * | * | * | 8 |
| Myers 2019 (2) |  | * |  | * | * | * | * |  | 5 |
| Friedman et al 2015 | * | * |  | * | * | * | * |  | 6 |
| Harkness et al 2018 |  | * | * | * | * |  | * |  | 5 |
| Satyanarayana et al 2021 |  | * |  | * | * | * | * | * | 6 |
| Shayegi-Nik et al 2024 |  |  | * | * | * | * | * |  | 5 |
