# Supplementary figures and images for: Syndemics and clinical impact of HIV and mental health conditions among people living with HIV: a systematic review and meta-analysis
Source: Front Public Health. 2026 Apr 10;14:1778334. doi: 10.3389/fpubh.2026.1778334 (PMC13137443; doi:10.3389/fpubh.2026.1778334)

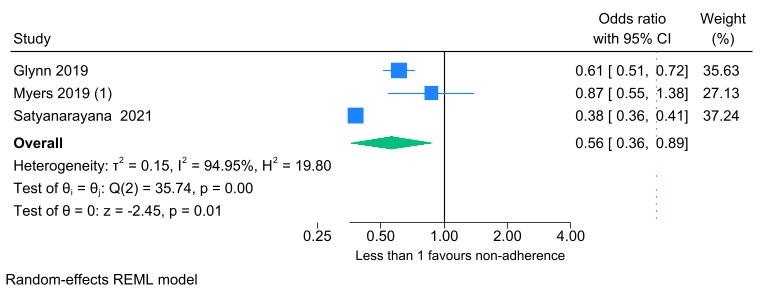

Supplement: Supplementary file 4 [file Image_1.jpeg]
